# Elderly Patients’ Perception of Previewing the Prosthetic Treatment Outcome

**DOI:** 10.25122/jml-2019-0125

**Published:** 2020

**Authors:** Mihaela Pantea, Ana Maria Cristina Tancu, Alexandru Petre, Marina Imre, Ecaterina Ionescu

**Affiliations:** 1. Department of Fixed Prosthodontics and Occlusology, Faculty of Dental Medicine, ”Carol Davila” University of Medicine and Pharmacy, Bucharest, Romania; 2. Department of Complete Denture, Faculty of Dental Medicine, ”Carol Davila” University of Medicine and Pharmacy, Bucharest, Romania; 3. Department of Orthodontics and Dento-Facial Orthopedics, Faculty of Dental Medicine, ”Carol Davila” University of Medicine and Pharmacy, Bucharest, Romania

**Keywords:** elderly, prosthetic preview, mock-up, temporary restorations

## Abstract

The demographic statistics indicate that, with the industrial development and the advances in the medical field, the average life of the population has started to increase significantly; consequently, the needs of treatments in geriatric dentistry are becoming more and more significant. The purpose of this study was to evaluate patients’ perception of diverse techniques used to obtain previews for final fixed prosthetic restorations: digital smile design (DSD), wax-up, indirect mock-up, temporary restorations, Computer-Aided Design (CAD). A focus group that included 11 participants, all of them geriatric patients, was formed; patients were invited to respond to a set of seven questions before and after various previsualization methods were applied. The focus-group report indicated that the indirect mock-up and the temporary restorations were considered by the geriatric patients as the best methods for obtaining good prosthetic previews. Comparing to the other tested methods, the use of indirect mock-up increased the acceptance of the prosthetic treatment plan and offered the most influential visual impact for 72.7% of participants. In addition, all tested previews allowed excellent communication with patients, the best results being obtained with the temporary prosthetic restorations (for 54.5% of participants). This approach represented a tool for better decisions regarding final dental restoration and has had a positive influence on patients’ acceptance of the treatment plan as well, making the final restoration more predictable.

## Introduction

The aging of the population is one of the most critical issues facing the globe today [1].

The United Nations (UN) reported that for the first time in history, in 2018, elderly persons (age 65+) outnumbered children under five years of age globally. Moreover, the World Health Organization (WHO) estimates that the number of persons aged 60 years and older will outnumber children younger than five years old by 2020. Therefore, the financial support of a numerically representative elderly population segment and the increasing demands in the medical services and social assistance field represent and will represent issues that modern societies must solve. Adoption of new modes of teaching geriatric dentistry and new models of dental practice to address the individual needs of elderly patients will be required [2, 3]. Strategies for providing better oral healthcare for elderly patients [4], personalized, individualized oral treatments and preventive geriatric dentistry should be implemented in order to assure healthy aging, as well; as a consequence of the above, the quality of life of older people will be improved.

It is estimated that the elderly population of “tomorrow” will be different from the elders of the past decades, as the former will be healthier, both physically and intellectually, more active, better educated, culturally literate, and more discerning consumers.

In this context, this paper presents several practical aspects related to modern prosthetic fixed restorations’ previews for elderly patients (age 65+); this subject is part of the current concerns of medical scientific research that aims at a deep understanding of the aging processes and finding ways to solve the problems faced by the elderly [5].

The aim of this study was to assess elderly patients’ perception of different methods used for the previsualization of planned fixed prosthetic restorations; patients’ perception was evaluated both before and after applying the respective methods, through a focus group. Moreover, the influence of the preview techniques on the acceptability of the proposed prosthetic treatment was analyzed to determine the preview method with the most significant impact on elderly patients.

## Materials and Methods

This study was initiated and coordinated at the Faculty of Dental Medicine, “Carol Davila” University of Medicine and Pharmacy, Bucharest, Romania and represents an updated fragment from the PhD thesis “Characteristics of the Fixed Prosthetic Treatment for Elderly Patients”, Mihaela Pantea, 2015. In this scientific research, a focus group consisting of 11 patients aged 65 and over, for whom we used one or more previsualization methods of the fixed prosthetic treatment: digital smile design (DSD), wax-up, indirect mock-up, temporary (interim) prosthetic restorations, Computer-Aided Design (CAD) was established. Two working sessions within the focus group were organized, the first one before, and the second after the practical (actual) application of the methods of previewing the results of the prosthetic treatments. The protocol for organizing and conducting the focus group consisted of a sequence of stages that have been adapted accordingly to the objectives of the present scientific research [6].

The selection criteria of the patients in order to participate in the present study were as follows: age 65 and over 65; legal and mental competence; ability to understand the proposed treatment; decision-making capacity; the necessity of fixed prosthetic treatments (supported on natural teeth or implants). Acceptance of participation in the study from patients in the focus group and written informed consent were obtained.

The established focus group was characterized by homogeneity (i.e., age 65 and over; patients in need of fixed prosthetic restorations); also, the traditionally recommended size of the focus group of 10 to 12 people was respected, as the group consisted of 11 participants.

For this study, a set of seven questions was established, which were applied in both focus-group sessions, before and after the actual implementation of the methods of previewing the results of the prosthetic treatments.

During the first meeting, all the selected previsualization methods were presented to each patient, including those corresponding to their specific clinical case. The patients received explications regarding the significance of each question and all the aspects they did not understand about the different previsualization techniques for prosthetic treatments, after which they were invited to answer the questions specially formulated for this study.

The questions aimed to evaluate the impact of these previsualization methods (DSD, wax-up, indirect mock-up, temporary prosthetic restorations, CAD) on the selected geriatric patients and the influence of these methods on the acceptance of the proposed treatment plan. The following set of questions was established:

1. Which method allows a better understanding - faster and clearer – of the proposed treatment plan: DSD, wax-up, indirect mock-up, temporary restorations, CAD?
2. Which method do you consider is able to make it easier for you to accept the treatment plan proposed by the dentist: DSD, wax-up, indirect mock-up, temporary restorations, CAD?
3. Which method was more demanding for you (in terms of physical effort, time, and others?
4. Which method had the strongest visual impact?
5. Which method do you consider to allow easier communication between you and the dentist (clear, fast, efficient)?
6. Do you consider that these preview methods are worth the effort?
7. Has your availability in being informed about the therapeutic solutions and the possibilities of their implementation increased?

During the second meeting, which was established after the actual application of one or more techniques of the previsualization of prosthetic treatments (suitable for each clinical case), patients were again invited to answer the set of questions.

The preview methods of the prosthetic treatments applied to the 11 elderly patients in the interval between the two focus group sessions are presented in the table below (Table 1): Digital Smile Design (coded as “DSD”), wax-up, mock-up, temporary restorations (coded as “Temp.”).

**Table 1:** Patients who took part in the study and the methods used for previewing the prosthetic treatment.

No.	Patient	Age	Sex	D.S.D.	Wax-up	Mock-up	Temp.	CAD
1	L.N	78	M	X	X	X		
2	J.E.	75	F	X	X	X		
3	M.E.	69	F	X	X	X		X
4	B.L.	76	F	X	X	X		
5	N.E.	72	F		X			
6	P.F.	69	F			X		
7	N.G.	66	F			X		X
8	E.D.	66	F				X	X
9	C.E.	76	F				X	
10	C.V.	65	M	X			X	X
11	B.V.	72	F	X	X		X	X

The patients answered the questions, both during the first meeting and the second meeting. The questions, as well as the answers and comments, were taken down and recorded by three dentists from the Faculty of Dental Medicine, the University of Medicine and Pharmacy “Carol Davila”, Bucharest.

The patients who agreed to continue the treatment were treated according to the proposed and accepted therapeutic solution.

Thus, by applying one or more preview methods to the 11 elderly patients, we obtained 6 previews for DSD, 6 previews for wax-up, 6 previews for mock-up, 4 previews for temporary restorations and 5 previews for CAD.

## Results

After the elderly patients found out in detail, during the first focus group session, about all the preview techniques for the planned prosthetic treatments included in the study (DSD, wax-up, indirect mock-up, temporary restorations, CAD) and after the discussions concerning their advantages and disadvantages, all 11 patients included in this study answered the questions.

Successively, one or more preview methods were applied for each patient, in compliance with the clinical situation, with the proposed treatment plan, with the patient's options and possibilities. The preview sessions were followed by a new session in the focus group, in which the photographs of the patients with the respective preview were analyzed, and the patients answered the questions again. The results obtained from the analysis of the patients’ answers to the questions asked in the focus group, both in the first session, marked with “I”, and in the second session, marked “II” are presented in Table 2.

**Table 2:** Participants’ answers in the two focus group sessions. “I” - first session and “II” - second session.

Question	DSD		Wax-up		Mock-up		Prov.		CAD	
I	II	I	II	I	II	I	II	I	II
Which method allows a better understanding - faster and clearer – of the proposed treatment plan: DSD, wax-up, indirect mock-up, temporary restorations or CAD?	3	1	2	1	4	5	0	3	2	1
Which method do you consider able to make it easier for you to accept the treatment plan proposed by the dentist: DSD, wax-up, indirect mock-up, temporary restorations or CAD?	2	0	0	0	7	8	0	2	2	1
Which method was more demanding for you (in terms of physical effort, time and others)?	8	6	0	0	3	4	0	1	0	0
Which method had the strongest visual impact?	2	0	1	0	6	8	2	3	0	0
Which method do you consider to allow easier communication between you and the dentist (clear, fast, efficient)?	2	0	2	0	3	4	3	6	1	1

Question	Yes		No		I do not know	
	I	II	I	II	I	II
Do you consider that these preview methods are worth the effort?	9	11	0	0	2	0
Has your availability in being informed about the therapeutic solutions and the possibilities of their implementation increased?	11	11	0	0	0	0

The major themes of the focus-group report highlighted that the mock-up method had the most significant impact on the geriatric patients, probably due to the possibility of direct visualization of the final result (the patients’ statements: “Really, this is how I would look like?” or “I look much younger” - these are examples that express the essence of the arguments for choosing a certain preview technique).

The mock-up method represented the preview method that allowed a better understanding of the treatment plan - both after the initial presentation of the techniques (for 36.3% of participants) and after the actual implementation (for 45.4% of participants).

The results also shown that the mock-up method made the proposed prosthetic treatment plan easier to be accepted (for 63.6% of participants in the first session and 72.7% in the second session) and it had the most significant visual impact as well (for 54.5% of participants in the first session and 72.7% in the second session). However, in elderly patients, the applicability of the indirect mock-up method in the frontal area has some limitations in the cases where patients present incorrect, defective prosthetic restorations (for example, over-contoured restorations with metal crowns placed in visible areas - the case of the female patient B.L.).

Most patients (54.5%) considered - in the second session of the focus group - that the communication with the doctor was facilitated when the temporary prosthetic restorations were made, compared to the first session, in which the temporary prosthetic restorations were equal to the mock-up method in terms of clarity of communication (for 27.2% of participants, corresponding to each method).

In the first session of the focus group, 72.7% of the elderly patients participating in the study considered that DSD required the most effort on their part.

Regarding CAD as a prefiguration method, the results shave shown that the elderly participants in this study do not exhibit a considerable interest on this matter: only 18.1% of participants (2 persons out of 11) considered that CAD allowed a better understanding of the treatment plan and increased its acceptance.

At the end of the first session of the focus group, most of the participants (9 out of 11, respectively 81.8%) believed that all the preview techniques are worth the effort, but after the previsualization was completed, all of the patients included in the study (100%) are convinced of the utility of this approach. Besides, the presentation of the preview techniques had increased the interest of all patients in being informed about the therapeutic solutions and their implementation.

## Discussions

In approaching the geriatric patient, the dental medical team had to exhibit an attitude corresponding to the bio-psycho-social complexity specific to the third age. The responsibilities of this team acquired new significance in the case of geriatric patients in terms of collaboration and communication with them, prefiguring and applying the actual therapeutic sequences, evaluating the preliminary and final treatment results, and monitoring patients [5].

In this study, we evaluated the perception of geriatric patients on various preview methods of planned dental prosthetic restorations, as well as the degree to which these methods influence the acceptance of the treatment plans by the patients. While DSD offers a two-dimensional prefiguration of the prosthetic results, simulating the final treatment result in the aesthetic area [7, 8], the traditional wax-up represents an objective 3D prefiguration of the future results of the prosthetic treatments [9], and the indirect mock-up represents the transposition of the wax-up into the buccal cavity of the patient before the irreversible changes on the tooth-periodontal structures begin [10]. On the other hand, the provisional/temporary restorations represent a mandatory stage in most clinical situations within the prosthetic rehabilitation, having the role of maintaining an oral, biological, mechanical and aesthetic balance until the final prosthetic restorations are performed, also allowing testing the functionality of the dento-maxillary apparatus. Regarding the Computer-Aided Design/Computer-Aided Manufacturing (CAD/CAM) technology, the computerized design has precise and fast results, currently being successfully included in designing the surgical and prosthetic treatment plan, in carrying out the prosthetic restoration project itself or the intermediate parts used during dental interventions [11].

The similarity between the answers offered by the patients participating in this study in the first and second sessions of the focus group confirmed a good understanding of the particularities of the different techniques for previewing the results of the final prosthetic treatments.

The mock-up method was the most valued preview method by the patients included in this study, in terms of understanding and acceptance of the prosthetic treatment plan. The direct visual impact of the mock-up increased the patient's motivation for treatment. It is known that for many older people, especially women, the completion of prosthetic rehabilitation (or failure to do so) has particular implications for their self-esteem and how the elder feels perceived by other people. DSD was considered the most demanding preview method (in terms of physical effort, time) for the participants in this study, probably due to the rather long photo sessions. However, patients understood the utility of DSD for wax-up and mock-up, both methods representing important stages in implementing the prosthetic treatment plan and the communication between dentists and dental technicians. The fact that the DSD method did not have such a significant impact is probably due to the lower interest in a preview method that does not give direct, tangible results.

Acceptance of the treatment plan by all elderly patients (after being presented the techniques of previewing the prosthetic treatments and after different methods were applied to each patient) indicated the importance of these preview methods.

In the context in which we are confronted worldwide with the visible numerical increase of older adults, the theme of this paper integrates into the current concerns of medical scientific research, which shows an increasing interest in the field of geriatric dentistry [12-16] and the implementation of the corresponding concepts in the university curricula [2, 3, 17, 18, 19]. The present study compares different methods of previewing the final prosthetic treatment outcome and their impact on the older people, representing a unique attempt of this kind, such approaches being absent in the scientific literature.

## Conclusions

Considering the limitations of this study, the following conclusions can be drawn:

1. The tested preview methods improved both the geriatric patients’ understanding of the proposed treatment plan and the communication between the doctor and the patient; the patients accepted the proposed treatment plan, although, for most of them, it implied complex, multidisciplinary rehabilitations, including endodontic, periodontal, surgical and prosthetic treatments;
2. The elderly patients’ favorite preview methods were the mock-up and the temporary restorations;
3. Elderly patients showed moderate interest in the Computer-Aided Design prefiguration method, but they understood its importance in making decisions about surgical and prosthetic treatments;
4. The presented preview methods, except for temporary restorations, are non-invasive and allow modifications according to the patients’ functionality and requirements, without affecting the integrity of the dental and periodontal structures;
5. The previsualization methods involve additional effort and costs that are, however, compensated by the beneficial guiding elements that support the implementation of the prosthetic treatment plan.

## Conflict of Interest

The authors confirm that there are no conflicts of interest.
